# Volumetric and MGMT parameters in glioblastoma patients: Survival analysis

**DOI:** 10.1186/1471-2407-12-3

**Published:** 2012-01-03

**Authors:** Georgios Iliadis, Vassiliki Kotoula, Athanasios Chatzisotiriou, Despina Televantou, Anastasia G Eleftheraki, Sofia Lambaki, Despina Misailidou, Panagiotis Selviaridis, George Fountzilas

**Affiliations:** 1 Department of Radiation Oncology, "Papageorgiou" Hospital, Thessaloniki, Greece; 2 Department of Radiation Oncology, Interbalkan Medical Center, Thessaloniki, Greece; 3 Department of Pathology, Aristotle University of Thessaloniki, School of Medicine, Thessaloniki, Greece; 4 Department of Neurosurgery, St Luke's Hospital, Thessaloniki, Greece; 5 Section of Biostatistics, Hellenic Cooperative Oncology Group, Data Office, Athens, Greece; 6 Department of Medical Oncology, "Papageorgiou" Hospital, Aristotle University of Thessaloniki, School of Medicine, Thessaloniki, Greece; 7 Department of Neurosurgery, "AHEPA" Hospital, Aristotle University of Thessaloniki, School of Medicine, Thessaloniki, Greece; 8 Department of Radiation Oncology, Interbalkan Medical Center, Asklipiou 10, 57001, Pylaia Thessaloniki, Greece

## Abstract

**Background:**

In this study several tumor-related volumes were assessed by means of a computer-based application and a survival analysis was conducted to evaluate the prognostic significance of pre- and postoperative volumetric data in patients harboring glioblastomas. In addition, MGMT (O^6^-methylguanine methyltransferase) related parameters were compared with those of volumetry in order to observe possible relevance of this molecule in tumor development.

**Methods:**

We prospectively analyzed 65 patients suffering from glioblastoma (GBM) who underwent radiotherapy with concomitant adjuvant temozolomide. For the purpose of volumetry T1 and T2-weighted magnetic resonance (MR) sequences were used, acquired both pre- and postoperatively (pre-radiochemotherapy). The volumes measured on preoperative MR images were necrosis, enhancing tumor and edema (including the tumor) and on postoperative ones, net-enhancing tumor. Age, sex, performance status (PS) and type of operation were also included in the multivariate analysis. MGMT was assessed for promoter methylation with Multiplex Ligation-dependent Probe Amplification (MLPA), for RNA expression with real time PCR, and for protein expression with immunohistochemistry in a total of 44 cases with available histologic material.

**Results:**

In the multivariate analysis a negative impact was shown for pre-radiochemotherapy net-enhancing tumor on the overall survival (OS) (*p *= 0.023) and for preoperative necrosis on progression-free survival (PFS) (*p *= 0.030). Furthermore, the multivariate analysis confirmed the importance of PS in PFS and OS of patients. *MGMT *promoter methylation was observed in 13/23 (43.5%) evaluable tumors; complete methylation was observed in 3/13 methylated tumors only. High rate of MGMT protein positivity (> 20% positive neoplastic nuclei) was inversely associated with pre-operative tumor necrosis (*p *= 0.021).

**Conclusions:**

Our findings implicate that volumetric parameters may have a significant role in the prognosis of GBM patients. Furthermore, volumetry could help not only to improve the prediction of outcome but also the outcome itself by identifying patients at high risk of treatment failure and, thus, seek alternative treatment for these patients. In this small series, MGMT protein was associated with less aggressive tumor characteristics.

## Background

Glioblastoma (GBM) is still one of the most uniformly fatal tumors. Although various radiotherapy techniques and altered fractionation schedules [1-6], as well as different chemotherapeutic regimens [7,8] have been implemented, the overall survival of glioblastoma patients remains rather poor, with a median survival of 12-18 months. A major problem concerning the study of these patients is the identification of robust prognostic parameters. Evaluation of prognostic factors is vital to improve research pursuing new therapies for glioblastomas, since a better randomization or stratification into various treatment arms can be achieved.

In brain tumor research, the importance of tumor size as one of them has long been debated. In contrast to other tumor sites (head and neck, ovary, uterine cervix, stomach), there are numerous studies [9-15] marking out tumor size as a prognostic factor or as a predictor of outcome of certain therapies.

Treatment of high grade gliomas, glioblastomas included, involves radiotherapy with concomitant administration of the alkylating agent temozolomide [16]. The addition of temozolomide seems to benefit patients with tumors exhibiting methylated CpG islands at the promoter and enhancer regions of the gene encoding for O^6^-methylguanine methyltransferase *(MGMT) *[17,18]. The assessment of *MGMT *promoter methylation is currently considered as mandatory for patient selection in clinical trials [19]; testing for *MGMT *methylation is nevertheless still not officially requested as a marker predictive for temozolomide response in clinical practice, mainly due to methodological shortcomings [20] and to the lack of alternative treatment options in patients without *MGMT *promoter methylation [16]. In addition, the prognostic significance of *MGMT *promoter methylation regardless of therapeutic intervention remains controversial [17,21].

In the present study, we explored the prognostic significance of several volumetric parameters, for overall survival and progression-free survival in patients harboring glioblastoma and treated postoperatively with radiotherapy and temozolomide. Our purpose was to decide if there is still a role of the volumetric MR (magnetic resonance) data in prognostic categorization of glioblastoma patients. This could be of crucial importance in designing future studies with more intensive therapeutic schemes. In addition, we compared MGMT related parameters with those of volumetry in order to observe possible implications of this molecule in tumor development and, subsequently, treatment response.

## Methods

### Patients

In this single-institutional prospective study, 65 patients older than 18 years of age with newly diagnosed and histologically proven glioblastoma (World Health Organization [WHO] grade IV astrocytoma), who attended clinics from July 2005 to August 2007 of Radiation Oncology in Papageorgiou General Hospital of Thessaloniki, Greece or Medical Oncology of Aristotle University of Thessaloniki in the same hospital, were enrolled in the study. Other eligibility criteria included: a) preoperative MRI scan b) WHO performance status of 2 or less c) adequate hematologic, renal, and hepatic function (absolute neutrophil count, ≥ 1500 per cubic millimeter; platelet count, ≥ 100,000 per cubic millimeter; serum creatinine level, ≤ 1.5 times the upper limit of normal in the laboratory where it was measured; total serum bilirubin level, ≤ 1.5 times the upper limit of normal; and liver-function values, < 3 times the upper limit of normal for the laboratory). The study was approved by the Ethics committee of Aristotle University of Thessaloniki, Greece and written informed consent was provided for every patient included.

### Treatment

All patients received radiotherapy and chemotherapy with Temozolomide. Radiotherapy was delivered using linear accelerators with energy of 6 and/or 18 MV and consisted of fractionated focal irradiation at a dose of 2 Gy per fraction given once daily five days per week over a period of six weeks, for a total dose of 60 Gy. All patients were treated supine and a thermoplastic mask was used as an immobilization device. For all patients we used CT simulation and the plan was performed with three-dimensional planning systems. Target volumes were based on postoperative MRIs. Two gross tumor volumes (GTV) were defined. The initial GTV (GTV1) was defined as T2 or FLAIR abnormality, including any enhancement in T1 and the surgical cavity, and the boost GTV (GTV2) as the contrast enhanced T1 abnormality, including the surgical cavity. The corresponding clinical target volumes (CTV1 and CTV2) and planning target volumes (PTV1 and PTV2) were generated by adding 2 cm on the GTVs to account for sub-diagnostic infiltration and 0.5 cm on the CTVs to account for variations in set-up and reproducibility, respectively. The initial target volume (PTV1) was treated to 44 Gy and afterwards the PTV2 for the rest 16 Gy, to a total of 60 Gy.

Chemotherapy with Temozolomide consisted of concurrent and adjuvant to radiation therapy phase in accordance with the EORTC 26981/22981; NCIC CE3 intergroup trial [22], with slight variations. After completion of 6 cycles of chemotherapy it was at the investigator's discretion to continue for 6 more cycles depending on response to the therapy.

### MRI acquisition and volumetry

All patients underwent pre- and postoperative MRI (magnetic resonance imaging) scans. The postoperative MRI scan was acquired one week before the initiation of radiotherapy and at least 21 days after surgery, as we waited for the acute postoperative abnormalities to subside.

For the purpose of volumetry, T1 and T2-weighted MR sequences were used. Since the MR scans were not available in an electronic format, but only in hard copies, they were digitized, by means of a commercial high-resolution scanner. Before determining tumor volume with our specialized software, images were converted to the widely used DICOM (Digital Imaging and Communications in Medicine) format with a different computer application. Our volumetric method was previously described in detail [23]. Briefly, the investigator contoured the volume of interest (VOI) on each MR slice. The software was able to calculate the volume of the VOI using the following formula:

V= ∑Si*z

**Figure 1:**
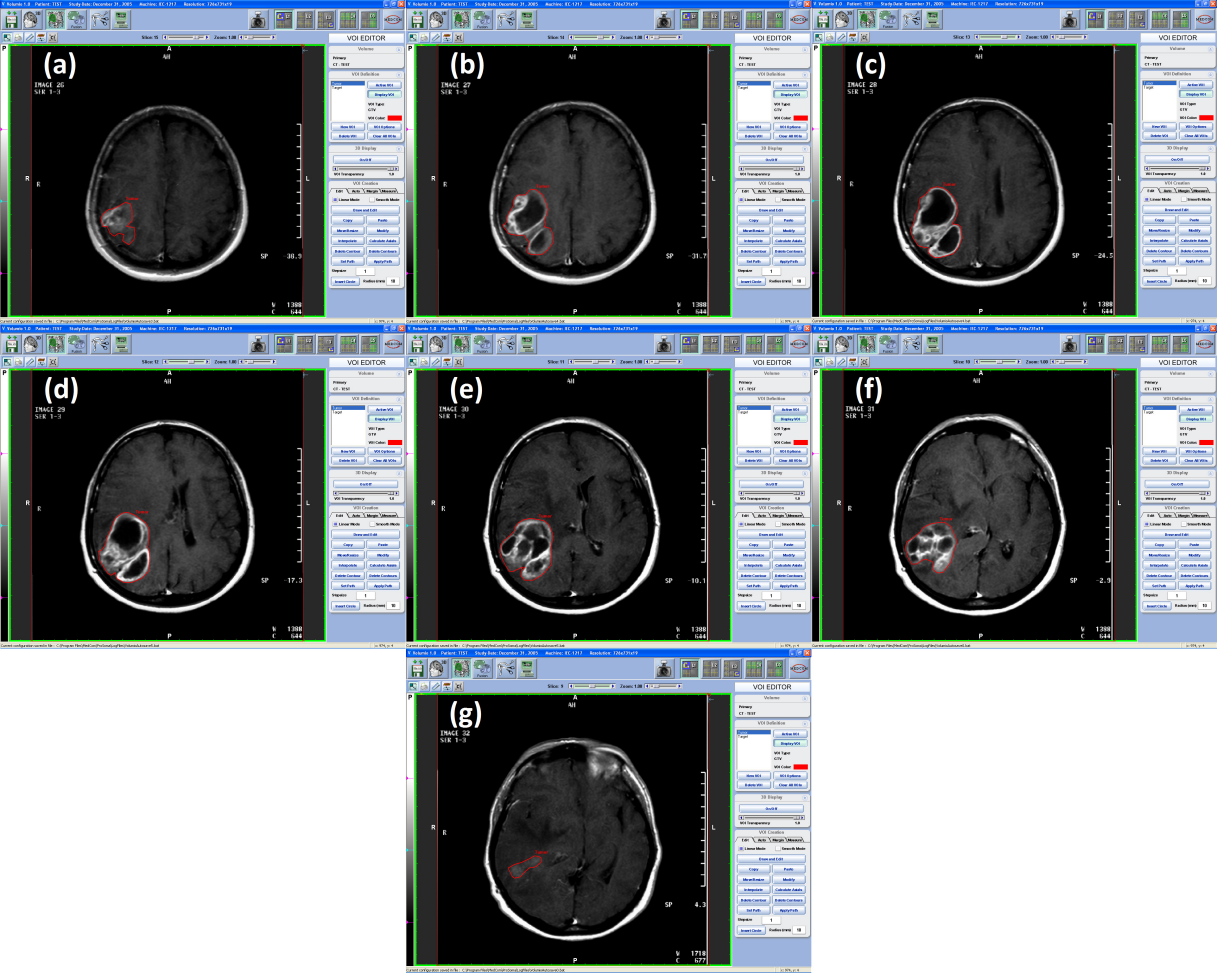
**(a-g)-Example of contouring**. Preoperative enhancing tumor in a glioblastoma patient.

**Figure 2:**
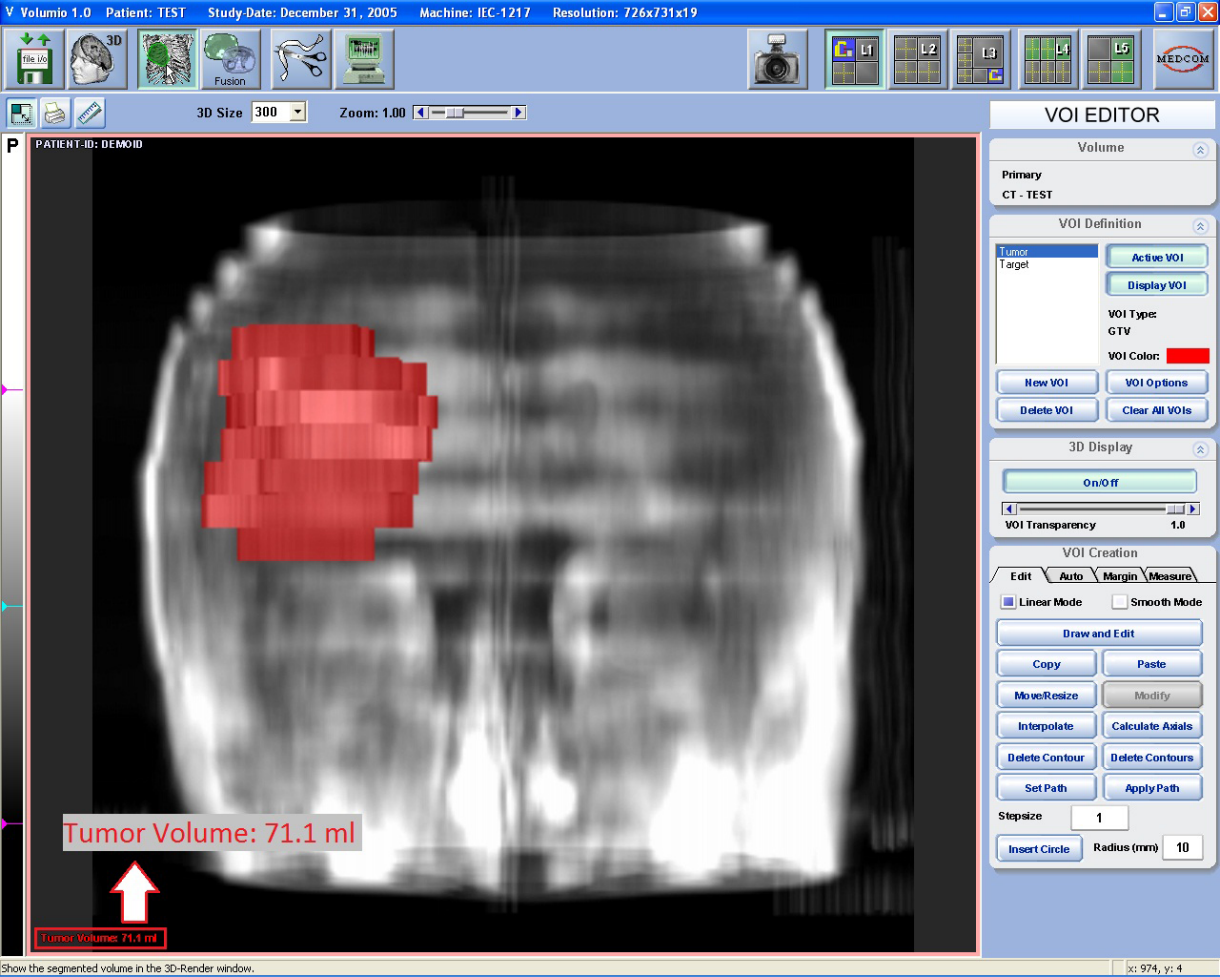
**Volumetry**. Three-dimensional reconstruction of the volume, based on the regions of interest contoured in Figure 1. The number in the left bottom corner represents the tumor volume in cc.

Where V is the volume of the VOI, S_i _the surface included by the contour of the VOI on each slice and z the slice thickness. The accuracy of this method is inversely proportional to slice thickness (Figure 1 and 2).

The volumes measured on preoperative MRIs were necrosis, enhancing tumor (including any region of central necrosis) and edema-T2 abnormality (including the tumor) and on postoperative ones-prior to radiochemotherapy (preRCT), net enhancing tumor. In the case of multifocal lesions, the sum of all measurable lesions was analyzed. All images were assessed by the same experienced radiation oncologist. Volumetry was performed before or during concurrent radiochemotherapy phase, as we tried to avoid bias from retrospective evaluation.

### Response evaluation and follow-up

During concurrent radiochemotherapy patients were evaluated weekly, clinically and with full blood counts and blood chemistry tests. Twenty-one to 28 days after the completion of the concurrent phase, patients underwent a comprehensive evaluation including radiologic assessment of the tumor with MRI. During adjuvant temozolomide therapy, patients underwent a monthly clinical evaluation and a comprehensive evaluation (including MRI) every 3 months until there was tumor progression or after two years of follow-up and every 3-4 months thereafter. The follow-up was updated on the May 4, 2010.

Tumor progression was defined according to MacDonald's response criteria [24]. When there was tumor progression patients were treated at their oncologist's discretion and the type of second-line therapy was recorded. Toxic effects were graded according to the National Cancer Institute Common Toxicity Criteria, version 3.0.

### *MGMT *promoter methylation status, MGMT mRNA and protein expression

Among the patients included in this study, formalin-fixed paraffin embedded (FFPE) material from stereotactic biopsies and partial or complete resections of the tumors was available for analysis for 44 patients (tumor tissue bank of the Hellenic Cooperative Oncology Group). The above tumors were used for Tissue MicroArray (TMA) construction (2 × 1.5 mm cores per tumor) and for DNA and RNA extraction.

DNA and RNA extraction were accomplished on manually microdissected whole sections at a total depth of 40 um for RNA and of 60 um for DNA. DNA was extracted with the QIAamp mini kit (Qiagen, Hilden, Germany) and RNA with Trizol-LS (Invitrogen, Paisley, UK) according to standard procedures. For first strand cDNA synthesis the Superscript III system with random primers and a final RNase H RNA removing step were employed (all from Invitrogen). DNA samples were normalized at 100 ng/ul, cDNA samples at 50 ng/ul; all samples were stored at -20°C until use. DNA quality was assessed with the multiplex PCR DNA control assay from BIOMED2 and RNA quality was evaluated with real time PCR (QRT-PCR) by employing a Taqman-MGB assay for beta-glucuronidase (*GUSB*) as endogenous control (assay ID: 4333767 F [Applied Biosystems]).

*MGMT *promoter methylation was evaluated with the Multiplex Ligation-dependent Probe Amplification (MLPA^®^) and the SALSA MS-MLPA KIT ME011 testing for methylation at three distinct CpG sites in the *MGMT *enhancer. The procedure and the evaluation of the results were accomplished according to the instructions of the manufacturer (MRC-Holland) and repeated twice to assess concordance of results (retention of the sample in the same category of methylation status). Evaluable results were yielded for 23/44 DNA tumor samples only (52.3%), all of which had amplification capacity for ≥ 300 bp, as revealed with the multiplex DNA control test.

*MGMT *gene expression was assessed by using a premade Taqman-MGB assay (Assay ID: Hs00172470_m1 [Applied Biosystems], ex 3-4, ref seq: NM_002412.3). Runs were performed for sample duplicates and readings were carried out at default settings in an ABI7500 real time PCR system equipped with the SDS v1.4 software. Upon initial control for cDNA amplification capacity, 41/44 samples yielded CT (cycle threshold) values ≤ 29 for the endogenous control amplicon (*GUSB*, as described above). Relative expression was assessed as the average 2^-dCT value (relative quantification value [RQ]) based on equal PCR efficiencies for very short amplicons [25] whereby dCT = *MGMT *CT-*GUSB *CT. Individual RQ values did not differ by more than 0.8 arbitrary units between duplicates.

Immunohistochemistry (IHC) for MGMT was performed on TMA sections of 2 um, using the monoclonal antibody MAB16200 (Chemicon, clone: MT3.1, anti-mouse), according to the instructions of the manufacturer (antigen retrieval: EDTA, dilution: 1:50). MGMT protein expression was evaluated with two different cut-offs, (a) 5% (absent ≤ 5%; present: > 5%) and (b) 20% (low ≤ 20%; high: > 20%) of tumor cells, respectively (modified after [17,26]).

### Statistical analysis

Categorical data are presented as frequencies and corresponding percentages, while continuous data are presented as median and range. 95% exact confidence intervals using binomial distribution for treatment responses are also given. For all measured volumes, exploratory analysis was performed using the quartiles of the corresponding distribution as predefined cut-offs, testing their distinguishing ability in patient's prognosis. For each cut-off, tumors were categorized in two categories (small vs. large volume). *MGMT *mRNA expression was assessed as a continuous and *MGMT *promoter methylation and protein expression as categorical variables.

Overall survival (OS) was measured from time of operation to patient's last contact or death. Progression free survival (PFS) was measured from time of operation to patient's last contact, disease progression or death from any cause without verified relapse. Time-to-event distributions were estimated using the Kaplan-Meier method and compared with the log-rank test. Univariate Cox regression analysis was performed to examine the prognostic significance of the examined volumetric or MGMT related parameters. In the multivariate Cox analysis a backward selection procedure with removal criterion *p *> 0.10, identified a subclass of significant variables among the following: Sex (male vs. female), age (< 50 vs > = 50), performance status (0 vs 1 or 2), type of excision (partial/biopsy vs subtotal vs total), pre-radiochemotherapy net enhancing tumor, preoperative necrosis, preoperative T2 abnormality, and preoperative enhancing tumor. For the categorisation of the type of excision we used data from the surgeons based on their impression during surgery and in some cases on postoperative CT scans. It was categorised as total, subtotal or partial/biopsy if there was resection of ≥ 99%, 75-99% and < 75% of tumor volume, respectively. For all tests, α = 0.05 level of significance was used. Analysis was conducted using SPSS 15.

## Results

Between July 2005 and August 2007, sixty-five patients (37 males, 28 females), with a median age of 59 years were assigned in our study. Patient's characteristics at the time of enrollment, along with tumor location, performance status and tumor volumetric parameters are shown in Table 1. All patients had surgical tissue diagnosis; 59 had undergone craniotomy and open biopsy followed by maximal feasible tumor resection (13 total resections, 33 subtotal resections and 13 partial resections or biopsies) and 6 received stereotactic biopsies.

**Table 1:** Selected patient and tumor characteristics

N = 65		
**Age (years)**		**Median (range)**

Median		59 (22-74)

		**N (%)**

< 50		15 (23)

≥ 50		50 (77)

**Sex**		

Male		37 (57)

Female		28 (43)

**PS**		

0		34 (52)

1		25 (38)

2		6 (9)

**Surgery**		

Partial/Biopsy*		19 (29)

Sutotal resection		33 (51)

Total resection		13 (20)

**Hemisphere**		

Left		33 (51)

Right		29 (45)

Bilateral		3 (5)

**Location**		

Temporal		20 (31)

Parietal		20 (31)

Occipital		3 (5)

Frontal		17 (26)

Deep		8 (12)

**Volumetric parameters**		**Median (range)**

	Enhancing tumor (cm^3^)	34.7 (2.3-117.5)

Preoperative	T2 abnormality (cm^3^)	108.1 (3.8-230.9)

	Necrosis (cm^3^)	5.8 (0-57)

PreRCT	Net-enhancing tumor (cm^3^)	11 (0-80.8)

* 6 cases of stereotactic biopsy are included

### Treatment delivery and toxicity

Treatment characteristics are shown in Table 2. One patient prematurely discontinued both radiotherapy and temozolomide due to severe hematologic toxic effects of concomitant treatment and eight other patients did not receive adjuvant temozolomide (2 due to death, 2 due to progressive disease, 3 due to non-fatal toxic effects and one due to patient refusal), although they had completed concurrent treatment. Hematologic complications were by far the most common. Overall, twenty-three patients (35%) suffered from grade 3 or 4 hematologic toxicity. More specifically, thrombocytopenia presented in 16 (25%), leucopenia in 13 (20%), neutropenia in 14 (22%) and anemia in 1 (2%) of the patients. Regarding moderate to severe non-hematologic complications, 3 patients presented with thromboembolic events and 3 other patients suffered from pulmonary infections, one of whom died as a consequence of pneumocystis carinii pneumonia.

**Table 2:** Treatment

Radiotherapy (RT)	
Median total dose (range)	60 (48-60)

Median number of fractions (range)	30 (24-33)


**Chemotherapy (CT)**	

· *CT concomitant with RT*	65 (100)

Total number of cycles delivered	417

Median number of cycles delivered (range)	7 (2-8)

· *Adjuvant CT (post RT)*	56 (86)

Total number of cycles delivered	343

Median number of cycles delivered (range)	6 (1-12)

**Treatment status**	**N (%)**

Completed	31(48)

Discontinuation	34(52)

PD	19(29)

Death	5(8)

Toxicity (non fatal)	5(8)

Refused to continue	4(6)

Clinical deterioration	1(2)

### Response and survival

Thirty-one patients (48%) responded to therapy; seven of them completely (11%, 95% Confidence interval [CI]: 4.4-20.9) and twenty-four partially (37%, 95% CI: 25.3-49.8). Twenty-seven patients had stable disease (42%, 95% CI: 29.4-54.4) and five developed progressive disease. Two patients were not evaluated for response due to early tumor death and patient refusal.

**Figure 3:**
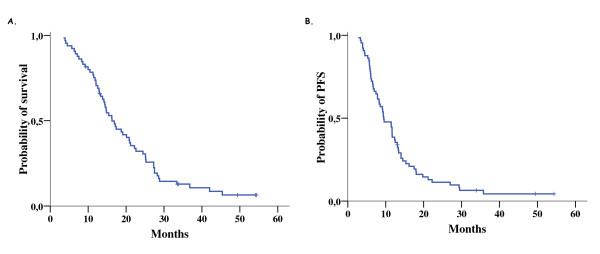
**Overall survival (OS) (A) and progression-free survival (PFS) (B)**.

After a median follow-up of 49.4 months, 61 patients experienced disease progression, 58 died and 2 patients were lost to follow-up. The median overall survival was 16.3 months (range 3.5-45.4 and 95% CI: 13.4-19.2) and the first and second year survival rate was 74% and 32%, respectively. The median progression-free survival was 9.5 months (range 2.8-35.8 and 95% CI: 6.8-12.1) and the first and second year progression-free survival rate was 38% and 11%, respectively. The corresponding Kaplan-Meier curves are depicted on Figure 3.

### Correlations of survival with clinical and volumetric parameters

In the univariate Cox regression analysis, sex, age and extent of surgery were not found to be statistically significant predictors for OS or PFS, whereas PS was found to influence both OS and PFS (Hazard ratio[HR] = 2.27, 95% CI:1.33-3.86; Wald's *p *= 0.003 and HR = 2.45, 95% CI:1.46-4.13; Wald's *p *= 0.001, respectively).

Concerning the volumetric measurements (using continuous values), preoperative T2 abnormality was not found to be a significant factor for survival or PFS, while for the other parameters, we identified a 2% excess risk of mortality for each one-unit increase in pre-radiochemotherapy net-enhancing tumor volume (HR = 1.02, 95% CI:1.01-1.04; Wald's *p *= 0.001) and a 2% excess risk of progression for each one-unit increase in preoperative necrosis volume (HR = 1.02, 95% CI: 1.00-1.05; Wald's *p *= 0.021). Moreover, a 1% excess risk of mortality and progression was observed for each one-unit increase in preoperative enhancing tumor volume (HR = 1.01, 95% CI: 1.00-1.02; Wald's *p *= 0.037 and HR = 1.01, 95% CI: 1.00-1.02; Wald's *p *= 0.041, respectively) (Table 3).

**Table 3:** Univariate Cox regression analysis of clinical factor and volume measurement with OS and PFS

	Overall Survival			Progression-free Survival		
	**HR**	**95% CI**	**p**	**HR**	**95% CI**	**p**

**Sex**						

Female	1			1		

Male	1.18	0.69-2.02	0.538	1.32	0.79-2.23	0.289

**Age**						

< 50	1			1		

≥ 50	1.54	0.83-2.89	0.174	1.43	0.77-2.67	0.259

**PS**						

0	1			1		

1 or 2	2.27	1.33-3.86	0.003	2.45	1.46-4.13	0.001

**Surgery**						

Partial/Biopsy	1			1		

Subtotal	2.02	0.95-4.32	0.069	0.96	0.53-1.73	0.881

Total	1.33	0.68-2.60	0.409	0.70	0.33-1.48	0.352

**Volumetry**						

PreOp Enhancing tumor	1.01	1.00-1.02	0.037	1.01	1.00-1.02	0.041

PreOp T_2 _abnormality	1.00	1.00-1.01	0.276	1.00	1.00-1.01	0.387

PreOp Necrosis	1.02	1.00-1.04	0.067	1.02	1.00-1.05	0.021

PreRCT Net-enhancing tumor	1.02	1.01-1.04	0.001	1.01	1.00-1.02	0.068

**Figure 4:**
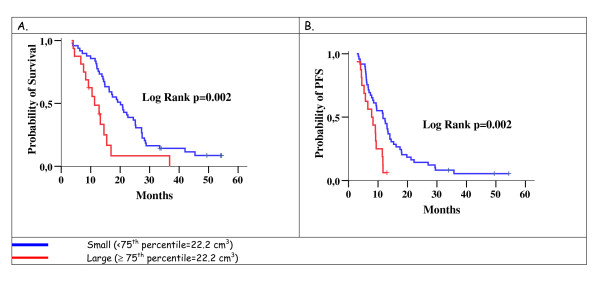
**Correlation of Overall survival (OS) (A) and progression-free survival (PFS) (B)**. Kaplan-Meyer curves for patients with large and small pre-radiochemotherapy net enhancing tumor volume.

Furthermore, in the exploratory analysis a prognostic threshold was detected for the preRCT net-enhancing tumor volume regarding both OS and PFS (75th percentile = 22,2 cm^3^). Patients with large remaining tumor postoperatively (preRCT net-enhancing tumor volume ≥ 22.2 cm^3^) had a reduced OS versus those with small remaining tumor (log-rank *p *= 0.002). The HR for large tumors was 2.59 (95% CI: 1.38-4.87, Wald's *p *= 0.003). In terms of PFS, large remaining tumors were associated with shorter PFS (log-rank *p *= 0.002) and the HR was 2.64 (95% CI: 1.38-5.02, Wald's p = 0.003) (Table 4 and 5 and Figure 4). For the rest of the volumes measured no significant association was found in terms of OS or PFS.

**Table 4:** Median OS and PFS according to pre-radiochemotherapy net enhancing tumor volume

	Events	Median	95% CI	Log-rank *p*
**Overall Survival**				0.002

Small (< 75th percentile = 22.2 cm^3^)	44/49	20.10	15.24-24.96	

Large (> = 75th percentile = 22.2 cm^3^)	14/16	11.28	6.96-15.60	

**Progression-free Survival**				0.002

Small (< 75th percentile = 22.2 cm^3^)	46/49	11.70	7.70-15.71	

Large (> = 75th percentile = 22.2 cm^3^)	15/16	7.84	4.37-11.31	

Log-rank test for overall and progression-free survival in association with large and small pre-radiochemotherapy net enhancing tumor volume

**Table 5:** Univariate Cox analysis of pre-radiochemotherapy net enhancing tumor volume with OS and PFS

	Overall Survival			Progression-free Survival		
	**HR**	**95% CI**	***p***	**HR**	**95% CI**	***p***

**PreRCT Net-enhancing tumor**						

Small (< 75th percentile = 22.2 cm^3^)	1	-		1	-	

Large (> 75th percentile = 22.2 cm^3^)	2.59	1.38-4.87	0.003	2.64	1.38-5.02	0.003

Univariate Cox analysis for association of large and small pre-radiochemotherapy net enhancing tumor volume with overall survival and progression free survival

Multivariate Cox regression analysis revealed that only performance status and preRCT net-enhancing tumor volume remained significant independent predictors of OS, while performance status and preoperative necrosis independently predicted PFS (Table 6). More specifically, preRCT net-enhancing tumor volume was associated with an increased risk for death (HR = 1.02, 95% CI: 1.00-1.04, Wald's *p *= 0.023). As expected, performance status of 1-2 was also associated with increased risk for death (HR = 2.01, 95% CI: 1.15-3.51, Wald's *p *= 0.014). Regarding PFS, preoperative necrosis and performance status were associated with increased risk for disease progression (HR = 1.02, 95% CI: 1.00-1.04, Wald's *p *= 0.030 and HR = 2.88, 95% CI: 1.66-5.01, Wald's *p *< 0.001 respectively).

**Table 6:** Multivariate Cox Analysis

	Overall Survival			Progression-free Survival		
	**HR**	**95% CI**	***p***	**HR**	**95% CI**	**Wald-*p***

**Age**						

< 50	1	-				

≥ 50	1.74	0.91-3.30	0.092			

**PS**						

0	1	-		1		

1 or 2	2.01	1.15-3.51	0.014	2.88	1.66-5.01	< 0.001

**Sex**						

Female				1		

Male				1.61	0.93-2.79	0.090

**PreOp Necrosis**				1.02	1.00-1.04	0.030

**PreOp Enhancing tumor**	1.01	1.00-1.02	0.066			

**PreRCT Net-enhancing tumor**	1.02	1.00-1.04	0.023			

### MGMT assessments in association with volumetric parameters and with patient outcome

Evaluation of *MGMT *mRNA expression was possible in 41/44 (93.2%), of MGMT protein expression in 35/44 (79.5%), and of *MGMT *promoter methylation status in 23/44 (52.3%) of the glioblastoma cases with available tissue blocks. RQ values for *MGMT *mRNA ranged from undetectable to 0.484 (mean: 0.028, ± SD: 0.075, median: 0.011). Ten out of 23 evaluable tumors (43.5%) were found methylated for at least one target with MS-MLPA (relative peak values > 0.25) and 13 tumors were unmethylated (relative peak values ≤ 0.25); only 3/10 methylated tumors (approximately 13% of all evaluable cases) were positive for all targets included in the assay with relative peak values > 0.75, i.e. the threshold set for the evaluation of complete methylation at the corresponding sites [27]. MGMT IHC results are shown in Table 7.

**Table 7:** MGMT immunohistochemistry

	n	%
*Evaluable results*	35/44	79.5

**MGMT absence (≤ 5% positive tumor cells)**	18/35	51.4

**MGMT presence (> 5% positive tumor cells)**	17/35	48.6

*Low MGMT expression (≤ 20% positive)*	11/17	64.7

*High MGMT expression (> 20% positive)*	6/17	35.3

Although *MGMT *methylated tumors showed lower mRNA expression, there was no statistically significant association between the results of the three methods. Neither *MGMT *promoter methylation nor *MGMT *expression (mRNA/protein) showed statistically significant association with patient PFS and OS.

In association with volumetric parameters, tumors with a higher rate of MGMT protein expression (> 20%, n = 6) were less necrotic at diagnosis than tumors with a lower rate of or negative for MGMT protein with IHC (n = 11) (Mann-Whitney *p *= 0.021). MGMT parameters were not associated with the rest of pre- or post-operative tumor volumetry parameters.

## Discussion

In the present study we explored the prognostic significance of several volumetric parameters for the progression-free and the overall survival in glioblastoma patients. All patients were treated with the same radiochemotherapy scheme, in order to ensure the uniformity of the population. Concerning the efficacy and toxicity of the radiochemotherapy scheme our results are in accordance with current literature [22,28].

As in most other studies dealing with glioblastoma patients [29-32], we confirmed the importance of the performance status as a factor influencing both survival parameters. It is of note, however, that the extent of resection was not found to be statistically significant. This may be explained by the subjectivity of this particular assessment (surgeons' impression and qualitative rough estimation of postoperative CT images) and dictates the need for the application of volumetry with early (within 48 h) postoperative MRIs. We can further assume, in this context, that the extent of resection may not be significant, when it is expressed as a percentage of the initial volume, but it is probably the absolute volume of the tumor remnant, which is the determinant factor affecting the prognosis.

The volumetric parameters, which were assessed in this study, were the pre-radiochemotherapy net-enhancing tumor volume and the preoperative enhancing tumor, necrosis and T2-abnormality. We found that pre-radiochemotherapy net-enhancing tumor volume and preoperative volume of necrosis are significant predictors of outcome. Our results are consistent with prior reports on the significance of volumetric parameters of glioblastomas.

In a recent study reported by Saraswathy et al. [33] the investigators evaluated the prognostic importance of MR markers (anatomic, perfusion, diffusion and metabolic) in pre-treatment (radiochemotherapy) scans in patients with GBMs. They found that all of the above parameters were associated with survival. In particular, the volume of contrast enhancing lesion was inversely correlated with survival.

In another study by Keles et al. [34] the authors concluded that the volume of residual disease (VRD) at the beginning of chemotherapy was a significant predictor for both survival time and time to progression in patients with recurrent glioblastomas. These patients, however, were neither chemotherapy-naive, nor newly diagnosed. In a different volumetric study of the same group [35] evaluating the effect of extent of tumor resection and VRD on survival, the authors reported that both of these parameters influenced significantly TTP and OS in patients with glioblastoma. This patient group is not uniform, though, as they did not all receive chemotherapy and the protocols are not noted. In addition, the volumetric measurements were not only performed in MR scans but CT images, so the results are not really comparable.

On the contrary, in the multivariate analysis of Tralins et al., investigated the use of 18 F-FDG PET for the guidance of radiation dose escalation in GBM patients, it was found that only the volume of uptake and not the volumes measured on MR images, including the T1-weighted gadolinium enhancement, were of prognostic significance for survival or time to progression [36]. That disagreement with our results could be explained by the different time of MRI acquisition, since in the above study it was performed during the course of radiotherapy.

In a recent study of Cao et al. [37] the authors concluded that only the vascular leakage volume measured 1-2 weeks prior radiation therapy in dynamic contrast enhanced T2*-weighted images is of predictive value for survival and not the volume of contrast enhanced lesion measured in T1 weighted (nondynamic) images. However, in this study only patients with residual tumor volume > 4 cm^3 ^were included and not all of the patients received chemotherapy and that could explain the discrepancy with our results.

At this point, we have to note that the prognostic value of contrast enhancement has certain limitations. This has been illustrated in a recent study by Piroth et al. [38], correlating the volumetric findings of MRI with those of positron emission tomography (PET) using O-(2-[(18)F]fluoroethyl)-L-tyrosine (FET) and concluding that the latter has a stronger prognostic impact. To summarize, although our study shows that pre-RCT net-enhancing tumor has a prognostic value, the results from the literature are rather contradictory in this context.

Concerning the influence of preoperative enhancing tumor volume, although it was found, in univariate analysis, to exert a negative impact on OS and PFS, this effect disappeared when adjusted for other significant predictors in the multivariate analysis. Our results are almost identical with the results of Weber et al. [39] in a study evaluating the prognostic factors in cerebellar GBM. Similarly, in a large retrospective study from Lacroix et al. [29] the multivariate analysis of 416 patients with GBM did not show any correlation of preoperative tumor volume with survival. There are numerous other studies [40-42] supporting the lack of prognostic importance of the preoperative enhancing tumor volume. On the contrary, Xue et al. concluded [43] that the accurate preoperative measurement of tumor volume with computer-based three-dimensional reconstruction is an important prognostic factor in high-grade gliomas. The description of the regions of interest that were delineated is not mentioned (contrast enhancement, margins, edema etc.), and additionally, the population of the study is not uniform, comprising anaplastic astrocytomas and glioblastomas altogether.

The most logical explanation for the difference in prognostic significance between pre and postoperative enhancing volume is the variation of the extent of resection. Since extent of the resection is not primarily correlated with the volume of the tumor, but with other factors as well (e.g. location, infiltration of critical areas), there is no distinct correlation between those two volumes. We also have to take into consideration that a variable period of time usually intervenes between the operation and the initiation of treatment, during which tumor re-growth may occur, thus rendering the pretreatment volume the most decisive factor for survival.

Necrosis is the imaging hallmark of GBM [44] and is believed to indicate rapid growth and malignant behavior [45]. We found that the volume of preoperative necrosis is a significant independent prognostic factor that negatively affects progression free survival, confirming several earlier studies pointing out this negative impact [29,42]. In this context, it was interesting to observe that tumors with a high rate of MGMT protein positive tumor cells were significantly less necrotic, a finding that may be related to the recently shown decreased tumorigenicity of MGMT expressing cells in preclinical models [46]. However, there are recent volumetric studies that failed to show any correlation between the absolute volume of necrosis and survival [47-49]. Our finding suggests that large volume of necrosis is indicative of a more aggressive phenotype, which is also in accordance with the well established pathological view that large necroses are associated with sinister prognosis. Moreover, the necrotic core is associated with hypoxia, which has been shown to be a factor of poor response to radio or chemotherapy, possibly due to up-regulation of vascular endothelial growth factor (VEGF) expression that stimulates angiogenesis [50-54]. To our knowledge there is no volumetric study correlating the absolute volume of necrosis measured in preoperative MR scan in glioblastoma patients with PFS. Interestingly there was no correlation with overall survival. This could be partially explained by the different therapies following recurrence, as many of them contained anti-VEGF agents, which would be more beneficial for tumors with high VEGF levels [55].

The last preoperative volume measured was the T2 abnormality (high intensity signal in T2-weighted MR sequence). That volume includes the necrotic core, the enhancing tumor and a perimetric zone, consisting of vasogenic edema and tumor cells [56,57]. In our study there was no association between that volume and PFS or OS. This is in accordance with the results of Crawford et al. [47] and Li et al. [49]. That result seems straightforward since that volume includes a great amount of edema, which is correlated with the dose of the prescribed corticosteroids and, consequently, is not a reliable measure of tumor burden.

Our study, certainly, points out that the precise volume determination of anatomic parameters is still essential in brain tumor research. It is simple, accurate, cost-effective and easily applicable from most oncological specialties. There are also several other imaging modalities (perfusion and diffusion weighted MRI, proton MR spectroscopic imaging, PET-CT) which can play a more significant role, maybe more important than the volumetry of anatomic lesions [33,36,58-60]. More specifically, the use of amino acid tracers in PET has been shown in recent studies to be superior compared to MRI both in planning volumetric resection [61], as well as in predicting the outcome of glioblastoma patients prior and after RCT [38,62]. In addition, the significant correlation of the pretreatment volume of enhancement with overall survival, leads us to conclude that shortening (no longer than 6 weeks) of the interval between surgery and initiation of radiochemotherapy could be of great importance to survival, since it overcomes the problem of tumor regrowth [63]. This interval however, in the light of data presented by Blumenthal et al. [64], should not be earlier than 4 weeks allowing enough time for recovery of the brain from the surgical injury and edema.

There are certainly some drawbacks that we need to mention. As it has been pointed out in our previous study [23], the lack of homogeneity in the MRI scans, as well as the process of digitization, may cause several variations in tumor delineation and the subsequently determined volumes. In addition, as the MRI was not performed during the optional time window of the first 48 h after surgery, visualization of the enhancing lesion was probably affected by postoperative changes. Moreover, our analysis was based on a small sample size, which may invoke the usual statistical uncertainties.

In this study, we did not observe any association of MGMT related parameters with patient outcome. It should be noticed, however, that the MLPA method used to determine *MGMT *promoter methylation yielded evaluable results in only half the samples available for this investigation; the method works on paraffin tissue extracts but it requires relatively preserved DNA, which we only could obtain in a limited number of cases, as evaluated by a multiplex control DNA PCR assay. In comparison to the usually applied MSP-PCR, MLPA has the advantage of providing information on the methylation status of multiple sites in the *MGMT *regulatory region in a semi-quantitative manner [27]. The degree of this epigenetic change seems to be of predictive value, since tumors with incomplete *MGMT *promoter methylation are reported to fail on temozolomide, while those with complete methylation show a significant trend to respond to this treatment [65]. The rate of tumors with complete methylation in our series was comparable to this report, but statistics could not be performed since the absolute number of cases was low. These results, as well as the lack of concordance among *MGMT *promoter methylation status, mRNA and protein expression results, once again reflect the problems encountered when assessing MGMT status on routine histologic material, as already reported in previous comparative studies [66].

## Conclusions

Our study showed that the volume of residual net-enhancing tumor prior to radiochemotherapy significantly affects survival in glioblastoma patients, although relevant literature data are inconsistent. Additionally, the volume of preoperative necrosis seems to be of prognostic significance for the PFS. The other preoperative volumetric parameters studied (enhancing tumor, T2 high signal abnormality) did not significantly affect either OS or PFS. It is obvious that volumetry can still play a significant role in defining patients who run a greater risk if treated with conventional therapy (radiotherapy plus temozolomide). For these patients, alternative treatments should be sought and better stratification for future studies could be achieved. A high expression rate of MGMT protein in glioblastomas may be related to a more indolent disease phenotype.

## Competing interests

The authors declare that they have no competing interests.

## Authors' contributions

GI was the primary investigator and wrote the manuscript and has contributed in the enrolment of patients and treatment, VK carried out the molecular genetic studies and contributed to the writing of the manuscript, AC has been involved in drafting the manuscript, DT evaluated IHC stains, AE performed the statistical analysis, SL has contributed to the acquisition of data and the therapeutic management, DM and PS has contributed to the initial design of the study, the enrolment of patients and treatment, GF has made substantial contributions to the conception and design of the study and to the enrolment of patients and the treatment. All the authors have given final approval of the version to be published.

## Pre-publication history

The pre-publication history for this paper can be accessed here:

http://www.biomedcentral.com/1471-2407/12/3/prepub
